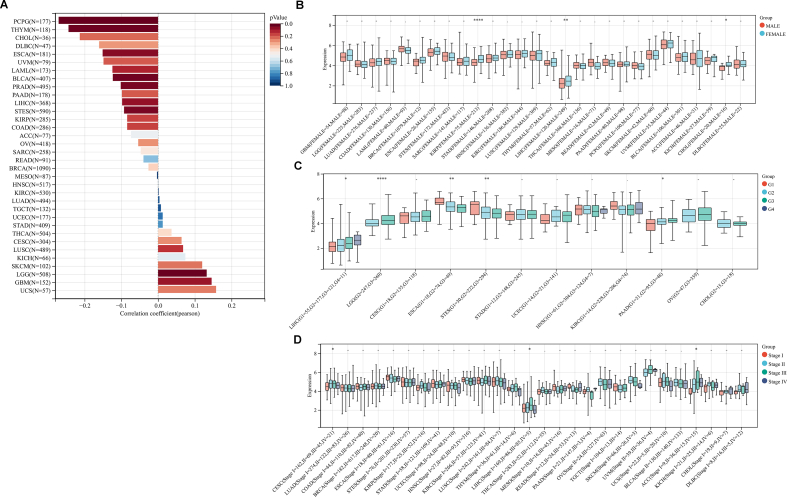# Pan-cancer landscape of disulfidptosis across human tumors

**DOI:** 10.1016/j.heliyon.2024.e40122

**Published:** 2024-11-06

**Authors:** Kun Fang, Suxiao Jiang, Zhengjie Xu, Meng Luo, Changsheng Yan

**Affiliations:** a Department of Surgery, Yinchuan Maternal and Child Health Hospital, Yinchuan, 750001, China; b Department of Surgery, The First Affiliated Hospital of Harbin Medical University, Harbin, Heilongjiang, 150001, China

**Keywords:** Disulfidptosis, Pan-cancer, Prognosis, Immunotherapy, Immune microenvironment, GYS1

## Abstract

**Objective:**

Disulfidptosis is a newly discovered disulfide stress-induced cell death form. Clinical significance and biological mechanisms of disulfidptosis in human cancers need to be further elucidated. Thus, this study was designed to characterize pan-cancer landscape of disulfidptosis across human tumors.

**Methods:**

Multi-omics features (transcriptomics, genomics, and DNA methylation) of disulfidptosis genes were investigated in TCGA pan-cancer cohorts. A disulfidptosis score system was defined across human tumors via ssGSEA. The activity of classical oncogenic pathways and hallmarks of cancer as well as the infiltration of immunocyte subpopulations were estimated, respectively. Drug sensitivity was inferred, and immune checkpoint blockade (ICB) response was evaluated in an independent cohort IMvigor210. ACHN, CAL-27, and NCI-H23 cells were transiently transfected with GYS1 siRNAs, and cell apoptosis and proliferation were measured through TUNEL and EdU assays, respectively.

**Results:**

Aberrant mRNA expression and DNA methylation of disulfidptosis genes as well as their genomic alterations were found in human tumors. The disulfidptosis score was utilized for quantifying the activity of disulfidptosis, which enabled to estimate patient prognosis. The disulfidptosis score presented positive correlations to angiogenesis and EMT, indicating the role of disulfidptosis in mediating tumor malignant features. Moreover, the score was negatively linked with infiltrating immune and stromal cells in the immune microenvironment. In the ICB cohort, shorter survival time was observed in patients with high disulfidptosis score, indicating the potential of disulfidptosis score in influencing clinical benefits from ICB. Additionally, tumors with low disulfidptosis score exhibited higher sensitivity to a few small molecular compounds, e.g., Sabutoclax, PRIMA-1MET, BIBR-1532, and Elephantin. Knockdown of disulfidptosis gene GYS1 effectively hindered tumor progression.

**Conclusion:**

Collectively, our findings depict a pan-cancer map of disulfidptosis to inform functional and therapeutic research.

## Graphical abstract

**Figure:**
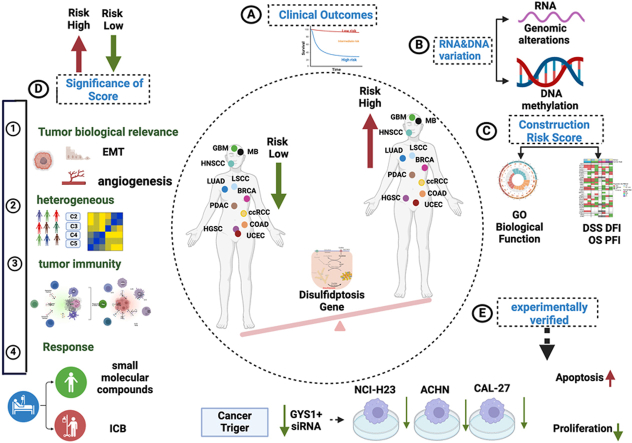

## 1 Introduction

Disulfides are relatively stable products maintaining the secondary, tertiary, and quaternary structures of proteins as inter- and intra-subunit cross-links, conferring physical and chemical stability to proteins, which can be produced in response to oxidative stress [1]. A latest study discovered a novel form of cell death elicited by disulfide stress, termed as disulfidptosis [1]. Unlike other forms of cell death, superfluous intracellular accumulation of disulfides in SLC7A11^high^ cells in the context of glucose starvation and without repair mechanisms leads to disulfide stress, contributing to disulfidptosis [2]. This cell death owns the main features of the collapse of cytoskeleton proteins and F-actin as a result of the intracellular accumulation of disulfides [3]. In addition, disulfidptosis requires three potential hallmarks: (i) high SLC7A11 expression that can import extracellular cysteine and export intracellular glutamate, leads to increased uptake of extracellular cysteine and superfluous intracellular accumulation of cysteine, thus provoking disulfide stress in cellular metabolism; (ii) abnormal disulfide bonds between actin cytoskeleton proteins are formed; (iii) a glucose starvation condition blocks glucose metabolism and generates the reduced form of NADPH through the pentose phosphate pathway (PPP) [4]. Once above conditions are met, disulfide is over-accumulated, eventually causing cell shrinkage and death [5].

As a key regulator of disulfidptosis, SLC7A11 has been evidenced to enable to protect tumor cells from oxidative stress, and presents overexpression in many cancers [[6], [7], [8]]. A recent study demonstrated that the level of SLC7A11 expression determines the sensitivity of cancer cells to oxidative stress, uncovering the context-dependent role of SLC7A11 in tumor biology [9]. Hence, disulfidptosis has the potential as a promising target for metabolic cancer therapy. However, clinical implication and biological mechanisms governing disulfidptosis in human tumors remain further exploration. Therefore, this study was conducted to characterize a pan-cancer map of disulfidptosis across human tumors. The discovery will potentially aid in the development of treatment strategies against cancer.

## 2 Materials and methods

### 2.1 Disulfidptosis-related gene set

The disulfidptosis-related gene set was summarized based upon a previously published study [1], containing SLC7A11, SLC3A2, RPN1, NCKAP1, NUBPL, NDUFA11, LRPPRC, OXSM, NDUFS1, and GYS1.

### 2.2 Acquisition of pan-cancer multi-omics data

Pan-cancer data were gathered from The Cancer Genome Atlas program (TCGA). The TCGA data sets were downloaded from USCS Xena (http://xenabroswer.net/hub) covering RNA sequencing (RNA-seq) (n = 23,088), patient information (n = 12,591), single nucleotide variation (SNV) (n = 10,680), copy number variation (CNV) (n = 10,680), and DNA methylation (n = 23,088) data across 33 tumor types for observations. Supplementary Table 1 displays the patient information.

### 2.3 Differential mRNA expression analysis

RNA-seq data were normalized by use of RSEM method. The mRNA expression levels were represented as normalized RSEM values. The fold change was calculated based upon mean (tumor)/mean (normal). P-value was estimated via a *t*-test. This analysis was implemented via “limma” R package (version 3.52.4) [10]. Pearson's correlation method was utilized for estimation of the correlation coefficients.

### 2.4 Survival analysis

Two survival analysis approaches: log-rank test of Kaplan-Meier and univariate-cox regression analyses were conducted for disease-free interval (DFI), disease-specific survival (DSS), overall survival (OS) and progression-free interval (PFI) utilizing “survival” R package (version 3.4.0). The P value < 0.05 was regarded as statistically significant.

### 2.5 Immunofluorescence (IF)

IF staining of SLC3A2 (HPA017980), RPN1 (HPA026828), NCKAP1 (HPA020449), NUBPL (HPA029203), LRPPRC (HPA036408), OXSM (HPA021293), NDUFS1 (HPA064605), GYS1 (HPA041598), SLC7A11 (HPA064215), and NDUFA11 (HPA072209) in cancer cell lines was acquired from The Human Protein Atlas (https://www.proteinatlas.org/). The subcellular localization of the disulfidptosis-related genes in the pan-cancer cell lines was explored by analyzing immunofluorescence staining data from the human protein atlas (HPA, https://www.proteinatlas.org/) database.

### 2.6 SNV analysis

SNV mutation frequency (percentage) of each gene's coding region was estimated following the formula: number of mutated samples/number of pan-cancer samples. SNV waterfall plots were generated utilizing “Maftools” R package (version 2.12.0) [11].

### 2.7 CNV analysis

CNV data were processed by use of GISTIC2.0 software [12], and the percentage of CNVs was calculated. The relationships between CNVs and mRNA expression were estimated via Pearson's product moment correlation coefficient and a t distribution test following merging RNA-seq and CNV data.

### 2.8 DNA methylation analysis

Methylation difference between tumors and normal tissues was defined via a student's *t*-test. Correlation of paired mRNA expression with DNA methylation was evaluated via Pearson's product moment correlation coefficient as well as a t distribution test.

### 2.9 Protein-protein interactions

The disulfidptosis gene set was uploaded onto the STRING online database (https://string-db.org/) [13]. Their protein-protein interactions were acquired by default parameters (interaction score >0.7), with subsequent establishment of the network.

### 2.10 Functional annotation analysis

“ClusterProfiler” R package (version 4.12.6) was adopted for conducting Gene Ontology (GO) and Kyoto Encyclopedia of Genes and Genomes (KEGG) enrichment analysis on disulfidptosis genes [14]. The results were visualized via circlize R package [15]. Pathways or functions with adjusted p value < 0.05 were considered as statistically significant.

### 2.11 Definition of a disulfidptosis score system

“GSVA” R package (version 1.44.5)was employed for single-sample gene set enrichment analysis (ssGSEA) analysis to define a disulfidptosis score system based upon the disulfidptosis gene set [16].

### 2.12 Z-score assessment of oncogenic pathways

The z-score algorithm was adopted for mirroring the activity of given pathways through integration of feature gene expression [17]. The gene sets of three oncogenic pathways: angiogenesis, epithelial-mesenchymal transition (EMT), and cell cycle that were acquired from the Molecular Signatures Database (MSigDB, http://software.broadinstitute.org/gsea/msigdb/) [18] as well as disulfidptosis were subjected to the z-score algorithm derived from GSVA package (version 1.44.5). The values of each gene set were enumerated as z-scores of angiogenesis, EMT, cell cycle, and disulfidptosis, respectively.

### 2.13 Hallmark gene set enrichment analysis

The gene sets of classical hallmark pathways were gathered from the MSigDB. Based upon the gene sets, the activity of the hallmark pathways was inferred via ssGSEA algotrithm using the “GSVA” R package (version 1.44.5) [19].

### 2.14 Immune microenvironment analysis

The relative infiltration levels of 28 immunocyte subpopulations in the immune microenvironment were inferred by use of ssGSEA on the basis of their signatures using the “GSVA” R package (version 1.44.5) [20]. Classical immune checkpoints were also gathered and analyzed.

### 2.15 Analysis of sensitivity of small molecular compounds

Based upon the Genomics of Drug Sensitivity in Cancer (GDSC) (https://www.cancerrxgene.org/) [21], the IC50 values of small molecular compounds were estimated for inferring the sensitivity by use of “pRRophetic” R package (version 0.5) [22]. Ten-fold cross-validation was subsequently implemented for the prediction accuracy evaluation.

### 2.16 Immune checkpoint blockade (ICB) response prediction

Infiltrating immune and stromal cells were estimated in tumors utilizing gene expression signatures through ESTIMATE computational method [23]. This study also collected and analyzed a real-world ICB cohort: IMvigor210 (http://research-pub.gene.com/IMvigor210CoreBiologies) that contained microarrays, prognostic outcomes, and anti-PD-L1 therapeutic response of metastatic urothelial cancer patients [24]. Kaplan-Meier analysis was utilized for assessing the survival probability in low and high disulfidptosis score groups, and log-rank test was accustomed to identifying the survival difference.

### 2.17 Cell culture and transfection

ACHN, CAL-27, and NCI-H23 were cultivated in Dulbecco's modified Eagle's medium (Invitrogen, USA) plus 10 % fetal bovine serum and 1 % penicillin-streptomycin in an incubator with 5%CO_2_ at 37 °C. Small interfering RNA (siRNA) against GYS1 (si-GYS1) and its negative control (si-NC) were synthesized by GenePharma (Shanghai, China). Under the manufacturer's specification, cells were cultivated in a 6-well plate, and when the70 %–80 % confluence, they were transfected by use of Lipofectamine 3000 (Invitrogen, USA). Following 48-h cultivation, the knockdown efficiency of GYS1 was evaluated.

### 2.18 Reverse transcription quantitative real-time (RT-qPCR)

Extraction of total RNA from tumor cells was achieved via Trizol reagent (Invitrogen, USA), with subsequent reverse transcription via HiScript II Q RT SuperMix for qPCR (Vazyme, Nanjing, China). Primer sequences included: GYS1, 5′-GCGCTCACGTCTTCACTACTG-3’ (forward), 5′-TCCAGATGCCCATAAAAATGGC-3’ (reverse); GAPDH, 5′-GGAGCGAGATCCCTCCAAAAT-3’ (forward), 5′-GGCTGTTGTCATACTTCTCATGG-3’ (reverse). RT-qPCR was implemented utilizing ChamQ/SYBR qPCR Master Mix (Vazyme, Nanjing, China). GYS1 expression was normalized to GAPDH with 2^−ΔΔCt^.

### 2.19 Terminal deoxynucleotidyl transferase dUTP nick end labeling (TUNEL) assay

TUNEL assay was achieved by use of TUNEL apoptosis kit (ATK00001; AtaGenix, Wuhan, China) under the manufacturer's procedures. Cell photographs were investigated utilizing a fluorescence microscope (BX53; Olympus, Japan). TUNEL-positive cells were defined as apoptotic cells, and the percentage of TUNEL-positive cells/DAPI-stained cells was estimated.

### 2.20 5-Ethynyl-2′-deoxyuridine (EdU) assay

8 × 10^3^ cells were cultivated into a 96-well plate. EdU staining was conducted utilizing BeyoClick™ EdU-594 cell proliferation assay kit (C0078S; Beyotime, Beijing, China) under the manufacturer's specifications. Cell images were acquired using a fluorescence microscope, and the percentage of EdU-positive cells/DAPI-stained cells was estimated.

### 2.21 Statistical analysis

All the analyses were implemented by in the R environment with version 3.5.2 and GraphPad Prism with version 9.0.1. The results are displayed as mean ± standard deviation if applicable. Student's t-test or Wilcoxon test was adopted for comparison between two groups, with one-way ANOVA test or Kruskal–Wallis test for comparison between multiple groups. Pearson's or Spearman's correlation methods were utilized for estimation of the correlation coefficients. P-value<0.05 was regarded as statistically significant.

## 3 Results

### 3.1 Landscape of transcriptional and prognostic features of disulfidptosis genes across pan-tumor types

Disulfidptosis genes presented transcriptional dysregulation in most tumor types (Fig. 1A and B). Supplementary Table 2 shows the abbreviations and full names of 33 cancer types. It was noteworthy that SLC7A11 exhibited distinct overexpression in most tumor types in comparison to matched normal tissues, especially CHOL. Based upon results from log-rank test and univariate-cox regression method, these disulfidptosis genes exhibited significant connections to patient survival (Fig. 1C and D). They acted as risky or protective factors dependent on tumor types. For instance, both methods indicated that GYS1 was a risky factor for KIRC, LGG, and MESO. In accordance with IF results, disulfidptosis genes: SLC3A2, RPN1, NCKAP1, NUBPL, LRPPRC, OXSM, NDUFS1, GYS1, SLC7A11, and NDUFA11 were broadly expressed in cancer cell lines: A-431, SiHa, U2OS, U-251MG, Hep-G2, PC-3, and Rh30 (Fig. 1E). The evidence suggested that disulfidptosis genes were aberrantly expressed in pan-cancer and linked with patient prognosis.

**Fig. 1:**
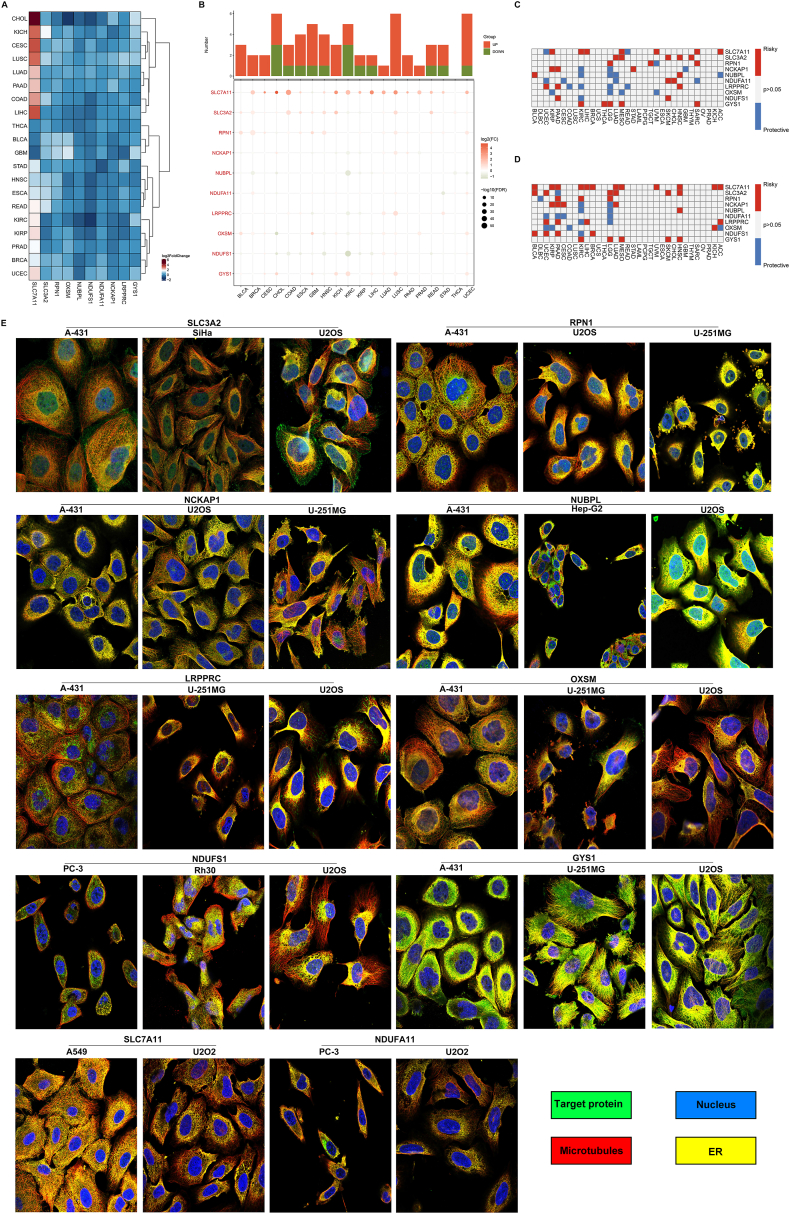
Landscape of transcriptional and prognostic features of disulfidptosis genes across pan-tumor types. (A) Heatmap illustrates the differential expression of disulfidptosis genes between tumors and corresponding normal tissues in each tumor type. (B) Bubble plots exhibit the down- or up-regulation of disulfidptosis genes in tumors versus matched normal tissues. (C, D) Heatmaps display the associations of disulfidptosis genes with patient survival through (C) log-rank test or (D) univariate-cox regression method. Blue, protective factor; red, risky factor; grey, no significance. (E) IF staining of disulfidptosis genes: SLC3A2, RPN1, NCKAP1, NUBPL, LRPPRC, OXSM, NDUFS1, GYS1, SLC7A11, and NDUFA11 in different cancer cell lines. Scale bar, 20 μm. (For interpretation of the references to color in this figure legend, the reader is referred to the Web version of this article.)

### 3.2 Genomic alterations and DNA methylation of disulfidptosis genes across pan-tumor types

**Fig. 2:**
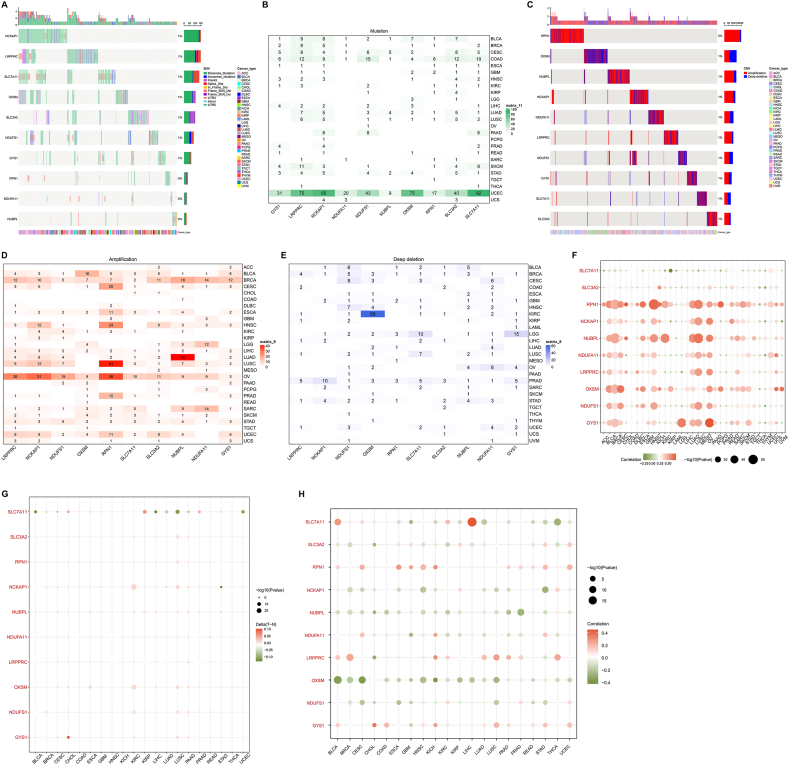
Genomic alterations and DNA methylation of disulfidptosis genes across pan-tumor types. (A) Waterfall plot illustrates the SNV frequency and type of disulfidptosis genes in pan-tumor types. Samples are ranked by tumor types. Genes are ranked by SNV mutational frequency. Distribution of mutational type is exhibited in the right panel. (B) Heatmap visualizes the SNV mutational frequency of each disulfidptosis gene across diverse tumor types. (C) Waterfall plot displays the CNV frequency. Blue, deep deletion; red, amplification. (D, E) Heatmaps show the amplification and deep deletion frequency of each disulfidptosis gene across distinct tumor types, respectively. (F) Bubble diagram exhibits the correlation between CNVs and transcript expression of disulfidptosis genes in each tumor type. Green, negative correlation; red, positive correlation. (G) Bubble diagram illustrates the differential methylation of disulfidptosis genes between tumors and matched normal tissues across pan-tumor types. (H) Bubble diagram shows the relationships between methylation and transcript expression levels of disulfidptosis genes in each tumor type. (For interpretation of the references to color in this figure legend, the reader is referred to the Web version of this article.)

The disulfidptosis genes: NCKAP1, LRPPRC, SLC7A11, OXSM, SLC3A2, NDUFS1, and GYS1 occurred somatic mutations in pan-cancer, with 1 % SNV frequency (Fig. 2A). Among tumor types, UCEC was found to present the most widespread somatic mutations of the disulfidptosis genes, such as SLC7A11 (92 %), NCKAP1 (85 %), LRPPRC (75 %), and OXSM (75 %) (Fig. 2B). The CNVs of the disulfidptosis genes also occurred across pan-cancer, with 2 % CNV frequency for RPN1, and 1 % CNV frequencies for OXSM, NUBPL, NCKAP1, NDUFA11, LRPPRC, NDUFS1, GYS1, SLC7A11, and SLC3A2 (Fig. 2C). Especially, RPN1 exhibited the most frequent amplification in LUSC, with the most frequent amplification of NUBPL in LUAD (Fig. 2D). Meanwhile, OXSM occurred the most frequent deep deletion in KIRC (Fig. 2E). It was thus evidenced that genomic alterations of the disulfidptosis genes presented broad heterogeneity in diverse tumor types. In addition, the CNVs of SLC7A11, and SLC3A2 were negatively linked with their expression in most tumor types, while the CNVs other disulfidptosis genes were positively linked with their expression (Fig. 2F), uncovering that the CNVs contributed to abnormal expression of the disulfidptosis genes. Aberrant DNA methylation of the disulfidptosis genes was also investigated in cancers, such as hypermethylation of GYS1 in CHOL, and hypomethylation of SLC7A11 in most tumor types (Fig. 2G). As expected, DNA methylation levels of the disulfidptosis genes displayed remarkably positive or negative associations with their expression in cancers (Fig. 2H), indicating the potential contributions of DNA methylation to their aberrant expression. Altogether, we infer that genomic alterations and DNA methylation both mediate the occurrence of disulfidptosis across pan-cancer.

### 3.3 Construction of a disulfidptosis score system for quantifying disulfidptosis in pan-cancer

**Fig. 3:**
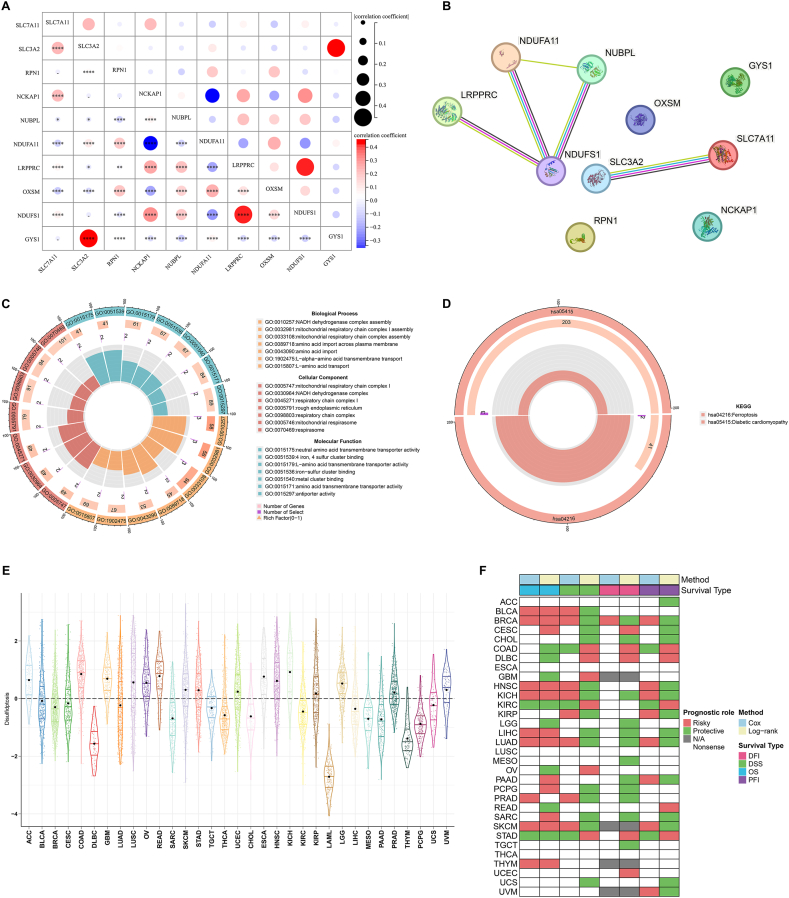
Construction of a disulfidptosis score system for quantifying disulfidptosis in pan-cancer. (A) Bubble chart illustrates the associations between disulfidptosis genes at the transcript levels. Blue, negative association; red, positive association. ∗p < 0.05; ∗∗p < 0.01; ∗∗∗∗p < 0.0001. (B) Protein-protein interactions among disulfidptosis genes. (C, D) Circle diagrams illustrate the main GO and KEGG terms enriched by disulfidptosis genes. (E) Distribution of the disulfidptosis scores across pan-tumor types. (F) Heatmap visualizes the associations between the disulfidptosis score and DFI, DSS, OS, and PFI in each tumor type through log-rank test and univariate-cox regression approach. (For interpretation of the references to color in this figure legend, the reader is referred to the Web version of this article.)

At the transcriptional level, the disulfidptosis genes exhibited the widespread relationships, such as GYS1 and SLC3A2; LRPPRC and NDUFS1 (Fig. 3A). From the protein-protein interaction perspective, LRPPRC, NDUFS1, NDUFA11, and NDUBPL were closely interacted (Fig. 3B). Additionally, an interaction between SLC3A2 and SLC7A11 was also observed. These interactions revealed the complexity of disulfidptosis process. GO enrichment results further proved the role of the disulfidptosis genes in the cell death mode, e.g., NADH dehydrogenase complex assembly, and mitochondrial respiratory chain complex assembly (Fig. 3C). Intriguingly, the disulfidptosis genes also participating in mediating ferroptotic cell death (Fig. 3D). Based upon the disulfidptosis genes, we proposed a disulfidptosis score system for quantification of disulfidptosis activity via ssGSEA (Fig. 3E). It was noted that disulfidptosis score was extensively heterogeneous across distinct tumor types. Notably, LAML presented the lowest disulfidptosis score. Next, this work assessed the prognostic significance of the disulfidptosis score. As illustrated in Fig. 3F, the disulfidptosis score exhibited the significant connections to patient survival outcomes: DFI, DSS, OS, and PFI, inferring that disulfidptosis participated in tumor progression.

### 3.4 Disulfidptosis score is positively linked with angiogenesis and EMT

**Fig. 4:**
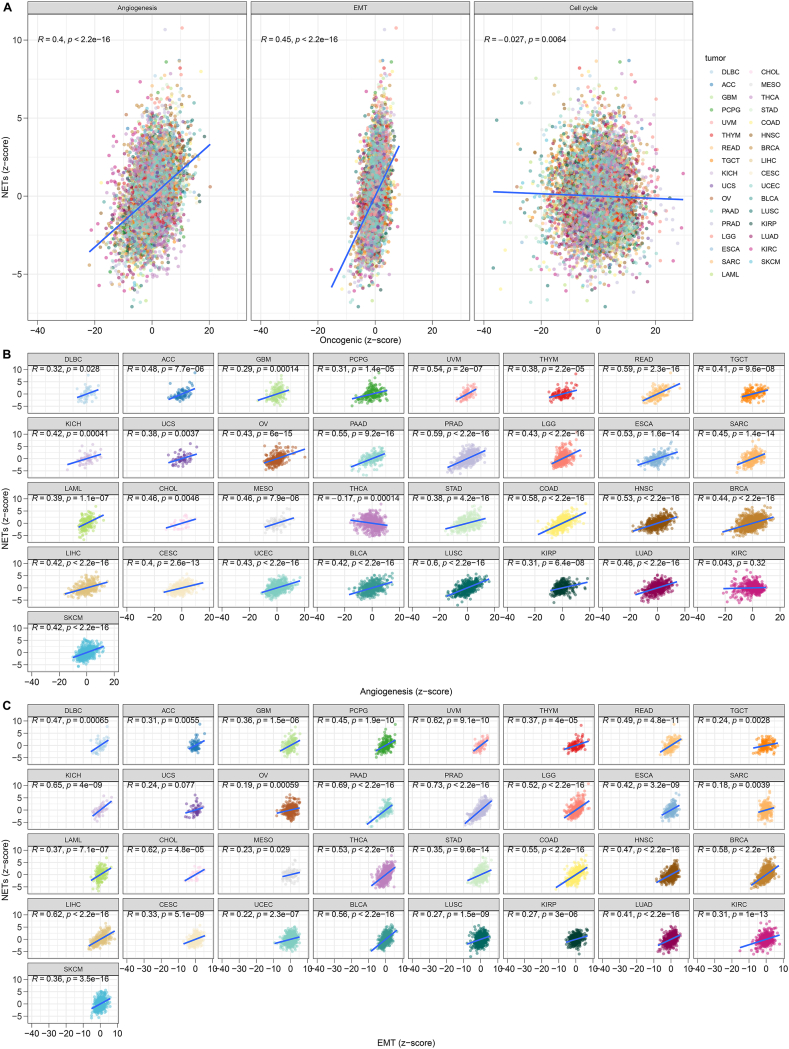
Correlation analysis on disulfidptosis score with classical oncogenic pathways: angiogenesis, EMT, and cell cycle in pan-cancer. (A) Scatter plots exhibit the relationships between the disulfidptosis score and angiogenesis, EMT, and cell cycle across pan-tumor types. Each tumor type is marked by unique color. (B) Scatter plots visualize the relationships between the disulfidptosis score and angiogenesis in each tumor type. (C) Scatter plots display the relationships between the disulfidptosis score and EMT in each tumor type. (For interpretation of the references to color in this figure legend, the reader is referred to the Web version of this article.)

In the process of dissimilation of normal cells to a malignant state, rapid proliferation, activated EMT, and angiogenesis are acquired, which are the hallmarks of cancer [25]. Among pan-cancer samples, the disulfidptosis score was found to be positively connected to angiogenesis and EMT (Fig. 4A). Nevertheless, it was observed that there was no significant connection between the disulfidptosis score and cell cycle. Furthermore, the positive connections between the disulfidptosis score and angiogenesis and EMT were found in nearly all tumor types (Fig. 4B and C). It was thus evidenced the role of disulfidptosis in participating in tumor aggressiveness and metastasis. Tumors with strong potential to facilitate disulfidptosis are possibly accompanied by more active angiogenesis in the tumor microenvironment as well as more aggressive tumor cells.

### 3.5 Disulfidptosis score is related to hallmark pathways and tumor immunity

**Fig. 5:**
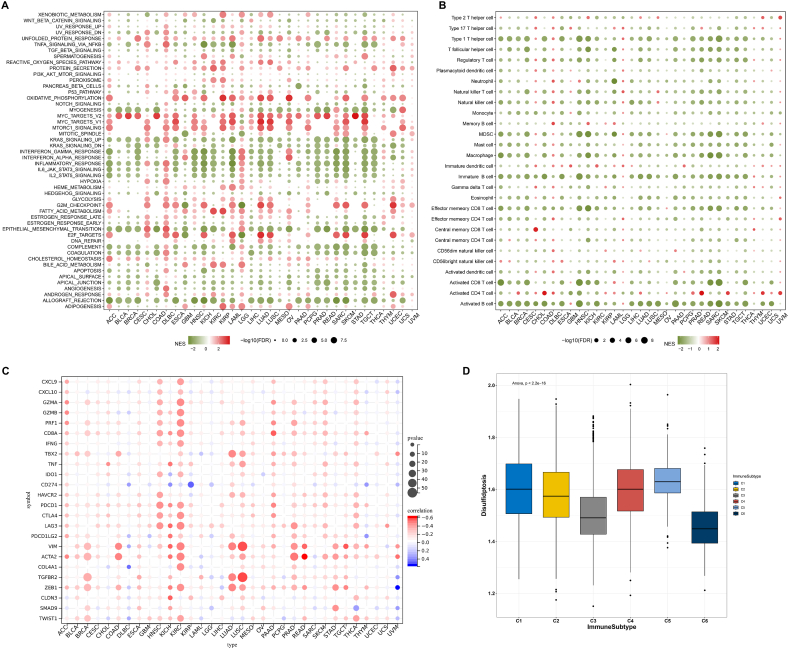
Associations between the disulfidptosis score and hallmark pathways, immune cells, immune checkpoints, and immune subtypes. (A) Bubble chart exhibits the relationships between the disulfidptosis score and hallmark pathways across diverse tumor types. Green, negative relationship; red, positive relationship. (B, C) Bubble chart displays the connections of the disulfidptosis score with immune cell populations, and immune checkpoint molecules. (D) Distribution of the disulfidptosis score across known immune subtypes. (For interpretation of the references to color in this figure legend, the reader is referred to the Web version of this article.)

The disulfidptosis score presented positive associations with multiple cancer hallmark pathways, e.g., MYC targets, mTORC1 signaling, and E2F targets, but presented negative associations with immune- or inflammation-related pathways, e.g., inflammatory response, interferon alpha/gamma response, IL2-STAT5 signaling, IL6-JAK-STAT3 signaling, allograft rejection, and complement (Fig. 5A). Moreover, the disulfidptosis score displayed negative relationships with most immune cell population across pan-cancer (Fig. 5B). As depicted in Fig. 5C, positive connections between the disulfidptosis score and classical immune checkpoints were investigated in diverse tumor types. Across pan-cancer, six immune subtypes have been defined to characterize intratumoral immune states: wound healing (C1), IFN-γ dominant (C2), inflammatory (C3), lymphocyte depleted (C4), immunologically quiet (C5), and TGF-β dominant (C6) [26]. The immunologically quiet (C5) subtype owned the highest disulfidptosis score; the TGF-β dominant (C6) subtype owned the lowest disulfidptosis score, followed by the inflammatory (C3) subtype (Fig. 5D), inferring that disulfidptosis might be connected to tumor immunosuppression.

### 3.6 Potential significance of disulfidptosis score in estimating responses of small molecular compounds and ICB

**Fig. 6:**
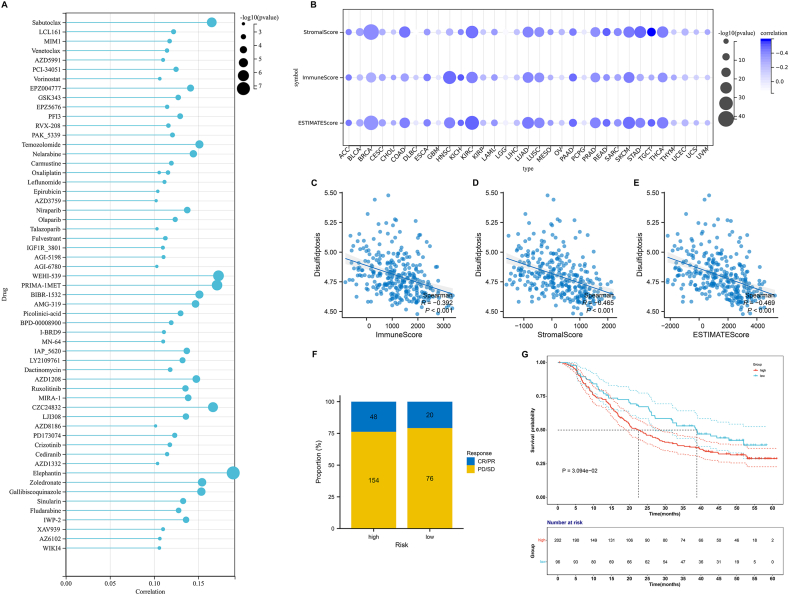
Relationships between the disulfidptosis score and responses of small molecular compounds and ICB. (A) Lollipop chart illustrates the connections of the disulfidptosis score with sensitivity of small molecular compounds. (B) Bubble chart visualizes the connections of the disulfidptosis score with immune, stromal, and ESTIMATE scores in each tumor type. (C–E) Scatter plots exhibit the relationships between the disulfidptosis score with immune, stromal, and ESTIMATE scores across pan-cancer. (F) Proportions of responders and non-responders to ICB in pan-cancer samples with low or high disulfidptosis score in the IMvigor210 cohort. (G) Survival probabilities of patients with low or high disulfidptosis score.

The disulfidptosis score was positively correlated to a few small molecular compounds, especially Sabutoclax, PRIMA-1MET, BIBR-1532, and Elephantin (Fig. 6A). Table 1 displays the pharmacological targets and involved pathways of these compounds. Across almost all tumor types, the disulfidptosis score presented negative connections to immune, stromal, and ESTIMATE scores (Fig. 6B–E), further uncovering that disulfidptosis might mediate infiltrating stromal and immune cells in tumors. Based upon the evidence, this study assessed whether the disulfidptosis score was connected to ICB response in the IMvigor210 cohort. Consequently, high disulfidptosis score group had the slightly lower proportion of responders to ICB (Fig. 6F). Nevertheless, patients with high disulfidptosis score owned the prominently shorter survival time versus those with low disulfidptosis score (Fig. 6G), inferring that the disulfidptosis score was connected to ICB resistance.

**Table 1:** The pharmacological targets and involved pathways of small molecular compounds related to disulfidptosis score.

Symbol	Target	Target pathway
Sabutoclax	BCL2, BCL-XL, BFL1, MCL1	Apoptosis regulation
LCL161	XIAP, cIAP1, cIAP2	Apoptosis regulation
MIM1	MCL-1	Apoptosis regulation
Venetoclax	BCL2	Apoptosis regulation
AZD5991	MCL1	Apoptosis regulation
PCI-34051	HDAC8, HDAC6, HDAC1	Chromatin histone acetylation
Vorinostat	HDAC inhibitor Class I, IIa, IIb, IV	Chromatin histone acetylation
EPZ004777	DOT1L	Chromatin histone methylation
GSK343	EZH2	Chromatin histone methylation
EPZ5676	DOT1L	Chromatin histone methylation
PFI3	Polybromo 1, SMARCA4, SMARCA2	Chromatin other
RVX-208	BRD4	Chromatin other
PAK_5339	PAK1, PAK2	Cytoskeleton
Temozolomide	DNA alkylating agent	DNA replication
Nelarabine	NA	DNA replication
Carmustine	NA	DNA replication
Oxaliplatin	DNA alkylating agent	DNA replication
Leflunomide	Pyrimidine synthesis inhibitor	DNA replication
Oxaliplatin	DNA alkylating agent	DNA replication
Epirubicin	Anthracycline	DNA replication
AZD3759	EGFR	EGFR
Niraparib	PARP1, PARP2	Genome integrity
Olaparib	PARP1, PARP2	Genome integrity
Talazoparib	PARP1, PARP2	Genome integrity
Fulvestrant	ESR	Hormone-related
Fulvestrant	ESR	Hormone-related
IGF1R_3801	IGFR1	IGF1R
AGI-5198	IDH1 (R132H)	Metabolism
AGI-6780	IDH2 (R14NAQ)	Metabolism
WEHI-539	NA	NA
PRIMA-1MET	NA	NA
BIBR-1532	NA	NA
AMG-319	NA	NA
Picolinici-acid	NA	NA
BPD-00008900	NA	NA
I-BRD9	NA	NA
MN-64	NA	NA
IAP_5620	IAP	Other
LY2109761	TGFB1	Other
Dactinomycin	NA	Other
AZD1208	PIM1, PIM2, PIM3	Other, kinases
Ruxolitinib	JAK1, JAK2	Other, kinases
MIRA-1	TP53	p53 pathway
CZC24832	PI3Kgamma	PI3K/MTOR
LJI308	RSK2, RSK1, RSK3	PI3K/MTOR
AZD8186	PI3Kbeta, PI3Kdelta	PI3K/MTOR
PD173074	FGFR1, FGFR2, FGFR3	RTK
Crizotinib	MET, ALK, ROS1	RTK
Cediranib	VEGFR, FLT1, FLT2, FLT3, FLT4, KIT, PDGFRB	RTK
AZD1332	NTRK1, NTRK2, NTRK3	RTK
Elephantin	NA	Unclassified
Zoledronate	NA	Unclassified
Gallibiscoquinazole	NA	Unclassified
Sinularin	NA	Unclassified
Fludarabine	NA	Unclassified
IWP-2	PORCN	WNT
XAV939	TNKS1, TNKS2	WNT
AZ6102	TNKS1, TNKS2	WNT
WIKI4	TNKS1, TNKS2	WNT

### 3.7 Knockdown of disulfidptosis gene GYS1 effectively hinders tumor progression

**Fig. 7:**
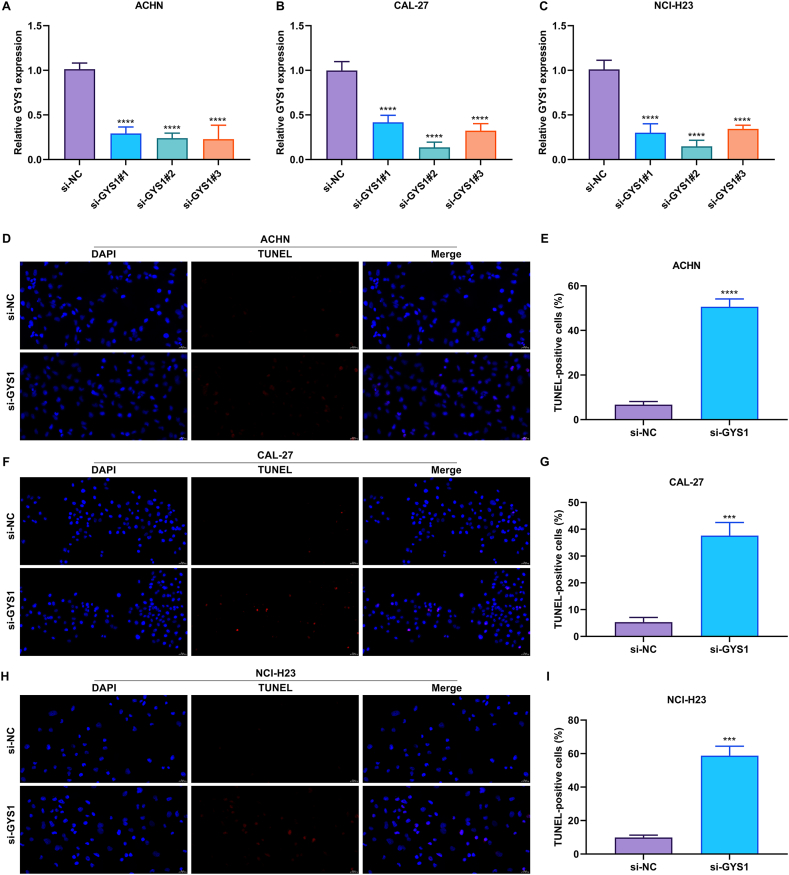
Knockdown of disulfidptosis gene GYS1 triggers apoptosis of cancer cells. (A–C) Verification of the knockdown effects of GYS1 by its specific siRNAs through RT-qPCR in three cancer cell lines: ACHN, CAL-27, and NCI-H23. (D–I) Representative photographs of TUNEL assay and quantification of the percentage of TUNEL-positive cells in ACHN, CAL-27, and NCI-H23 cells with si-GYS1 or si-NC transfection. Scale bar, 20 μm ∗∗∗p < 0.001; ∗∗∗∗p < 0.0001.

**Fig. 8:**
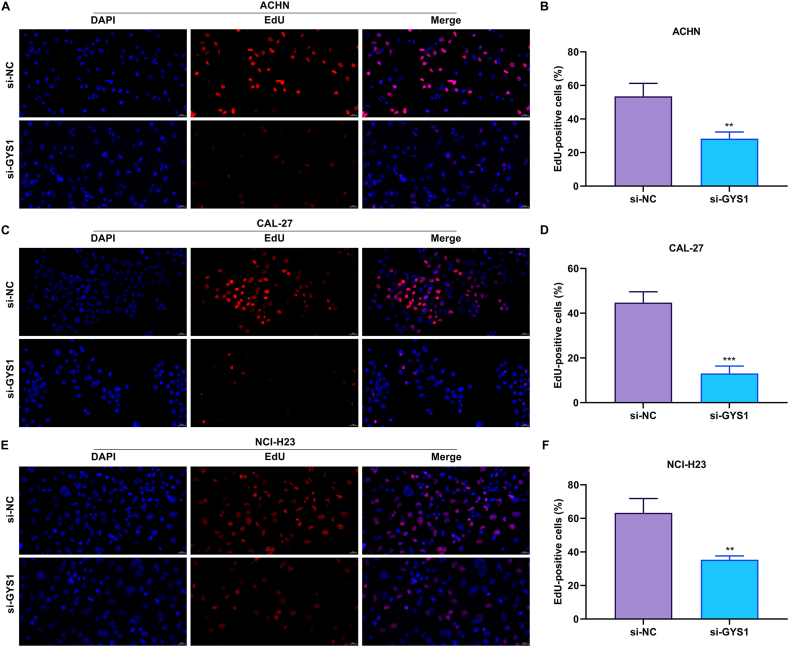
Knockdown of disulfidptosis gene GYS1 impairs proliferative abilities of cancer cells. (A–F) Representative photographs of EdU assay and quantification of the percentage of EdU-positive cells in ACHN, CAL-27, and NCI-H23 cells with si-GYS1 or si-NC transfection. Scale bar, 20 μm ∗∗p < 0.01; ∗∗∗p < 0.001.

Among the disulfidptosis genes, the role of GYS1 in tumor progression was further experimentally verified. GYS1 expression was remarkably suppressed through transiently transfecting si-GYS1 in three cancer cell lines: ACHN, CAL-27, and NCI-H23 (Fig. 7A–C). The knockdown of GYS1 was found to effectively trigger apoptosis of ACHN, CAL-27, and NCI-H23 (Fig. 7D–I). In addition, proliferative capacities of the three cancer cells were prominently attenuated in the context of GYS1 suppression (Fig. 8A–F). It was thus evidenced that GYS1 was a possible treatment target of human cancers. We further explored the function role of GYS1 in the Pan-cancers. As showed in Fig. S1, GYS1 was correlated with tumor progression-related pathways in multiple cancers. For example, in LGG and OV, GYS1 was correlated with IL2 stat5 signaling pathway, IL6 JAK stat3 signaling pathway, inflammatory response, and TNFA signaling via NF-kB signaling pathway. Moreover, in CESC, GBM, LGG, LIHC, LUAD, OV and PCPG, epithelial mesenchymal transition pathway were positively correlated with GYS1 expression.

In addition, we also investigate the relationship between GYS1 and clinical characteristics (age, gender, grade, and stage). The correlation analysis result revealed that GYS1 was negatively correlated with age in most tumors, including PCPG, THYM, CHOL, DLBC, ESCA etc. When comparing the expression of GYS1 between males and females, it was noted that in KIRP, LIHC, and CHOL, the levels of GYS1 were significantly higher in females than in males. Notably, we observed that GYS1 was significantly increased in the advance stage and grade in LIHC, indicating that GYS1 may act as a risk factor in LIHC and could potentially serve as a therapeutic target (Fig. S2).

## 4 Discussion

Disulfidptosis is a newly discovered disulfide stress-induced cell death form. Targeting disulfidptosis has been evidenced as an innovative treatment strategy against human tumors [27,28]. This work was designed to uncover clinical significance and biological mechanisms of disulfidptosis in human tumors.

We discovered that aberrant mRNA expression and DNA methylation of disulfidptosis genes as well as their genomic alterations occurred in human tumors. Especially, most disulfidptosis genes owned remarkable connections to patient prognosis across pan-cancer. Thus, in-depth research concerning disulfidptosis has crucial clinical significance. Based upon the complex and close interactions between disulfidptosis genes, we proposed the disulfidptosis score system enabling to quantify the activity of disulfidptosis in tumors. The disulfidptosis score presented remarkable connections to pan-cancer patient prognosis. This study shed light on a constitutive disulfidptosis-based signature that contributed to the progression of patients with pan-cancer. The disulfidptosis score divided all patients into low- and high-score groups. Due to the positive relationships between the disulfidptosis score and oncogenic pathways (EMT and angiogenesis) [29] and hallmarks of cancer, most tumor progression possibly occurred in high disulfidptosis-score samples.

Most drugs that enter clinical trials fail, usually owing to an incomplete comprehending of the mechanisms underlying drug responses [30]. Alterations in the cancer genome notably affect clinical responses to treatment and act as effective biomarkers for drug responses. Tumors with low disulfidptosis score were more sensitive to a few small molecular compounds, e.g., Sabutoclax, PRIMA-1MET, BIBR-1532, and Elephantin, indicating that disulfidptosis might influence drug sensitivity and resistance. Nevertheless, disulfidptosis-mediated biological mechanisms governing the drug responses require more investigations.

Immunologically quiet (C5) subtype presents the lowest response of lymphocytes, and the highest response of macrophages, mainly M2 macrophages. It was observed the highest disulfidptosis score in this subtype. Inflammatory (C3) subtype is characterized by up-regulated Th1 and Th17 genes, low to moderate proliferation of cancer cells, low aneuploidy and overall somatic copy number alteration levels [26]. TGF-β dominant (C6) subtype exhibits the highest TGF-β signature, high infiltration of lymphocytes as well as evenly distributed type I and type II T cells. The lowest disulfidptosis score was found in the C6 subtype, followed by the C3 subtype. Additionally, the disulfidptosis score was negatively connected to infiltrating immune and stromal cells in the immune microenvironment as well as was positively linked with classical immune checkpoints. Collectively, we inferred that disulfidptosis participated in mediating immunosuppression in tumors. In the independent ICB cohort IMvigor210, patients with high disulfidptosis score displayed worse survival outcomes. Together, this study defined disulfidptosis-based molecular subgroups of pan-cancer that potentially affected clinical benefits from ICB.

Among the disulfidptosis genes, it was experimentally evidenced that suppression of GYS1 effectively hindered tumor cell growth. It has been reported that GYS1 triggers glycogen accumulation and leads to tumor progression through activating NF-κB signaling [31]. GYS1 overexpression is connected to adverse outcomes and poor response to azacitidine in LAML [32]. Thus, targeting GYS1 represents a metabolic vulnerability of tumors [33]. Moreover, we also observed GYS1 was positively associated with higher grade and pathological stage patients in LIHC, suggesting that GYS1 have the potential to serve as indicator in prognosis or diagnosis of LIHC. Together, the disulfidptosis-based signature provides a possible method to assess the prognosis and therapeutic efficacy in pan-cancer patients, thus facilitating clinical practice and management. Nevertheless, the value of the disulfidptosis score in estimating pan-cancer prognosis and therapeutic response requires to be verified in prospective cohorts.

## 5 Conclusion

Altogether, our work provides the evidence for disulfidptosis in tumor progression, and probes out its potential in disease treatment. A deeper understanding of disulfidptosis in tumor lesions will provide a novel insight into fundamental cellular homeostasis and accelerate the development of innovative treatment against cancers.

## CRediT authorship contribution statement

**Kun Fang:** Writing – review & editing, Visualization, Methodology, Funding acquisition, Data curation, Conceptualization. **Suxiao Jiang:** Writing – original draft, Validation, Formal analysis. **Zhengjie Xu:** Writing – original draft, Validation, Formal analysis. **Meng Luo:** Writing – original draft, Visualization. **Changsheng Yan:** Writing – original draft, Supervision.

## Ethics approval and consent to participate

Not applicable.

## Consent for publication

The contents of this manuscript have not been copyrighted or published previously.

## Data availability statement

The original contributions presented in the study are included in the article/Supplementary Material. Further inquiries can be directed to the corresponding author.

## Funding

The research was supported by a Ningxia Reproductive Disease Clinical Medical Research Center Project (2023LCYX003), Ningxia Hui Autonomous Region Natural Science Foundation Project (2022AAC03748), Ningxia Hui Autonomous Region Natural Science Foundation Project (2021AAC03523), Basic research project of Yinchuan Maternal and Child Health Hospital (2022NYFYCX05).Yinchuan Science and Technology Innovation Project (2023SF25). Basic research project of Yinchuan Maternal and Child Health Hospital (2023NYFYCX01), Ningxia Young Top Talent Training Program (NRSF-2024-102),Yinchuan Academic and Technological Leader Reserve Program (YRCF-2024-5).

## Declaration of competing interest

The authors declare that they have no known competing financial interests or personal relationships that could have appeared to influence the work reported in this paper.

## Figures and Tables

**figs1:**
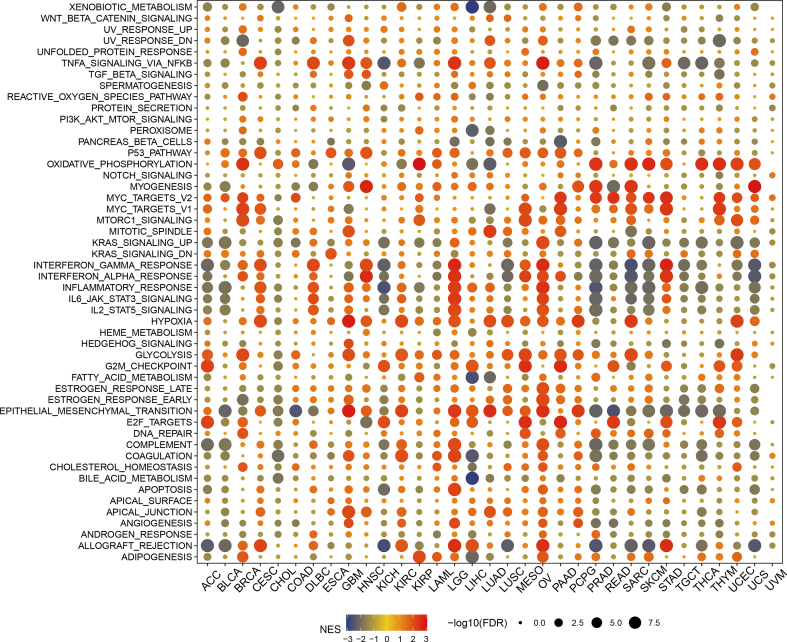


**figs2:**